# The J-shaped relationship between body roundness index and adult asthma: insights from NHANES 2001–2018

**DOI:** 10.3389/fnut.2025.1516003

**Published:** 2025-03-20

**Authors:** Kunpeng Sun, Yiyi Chang, Jing Jie, Chunyan Wang, Yue Gu

**Affiliations:** ^1^Department of Respiratory and Critical Care Medicine, First Hospital of Jilin University, Changchun, China; ^2^Department of General Medicine, First Hospital of Jilin University, Changchun, China

**Keywords:** asthma prevalence, Body Roundness Index, obesity, cross-sectional study, NHANES

## Abstract

**Background:**

Many studies have used Body Mass Index (BMI) to define obesity and examine its potential link to adult asthma. However, BMI overlooks body fat distribution, which may significantly impact health. Unlike BMI, the Body Roundness Index (BRI) can more accurately reflect body fat distribution. Therefore, this study examined BRI’s relationship with asthma prevalence in U.S. adults.

**Methods:**

This study was based on data from the National Health and Nutrition Examination Survey (NHANES) between 2001 and 2018 and covered 40,052 adult participants. Participants were categorized into four quartile groups based on their BRI levels: Quartile 1 (1.05, 3.80); Quartile 2 (3.80, 5.06); Quartile 3 (5.06, 6.61); Quartile 4 (6.61, 23.48). The association between BRI and asthma prevalence was assessed via weighted multivariate logistic regression, smoothed curve fitting, threshold effects, subgroup, and sensitivity analysis. BRI’s predictive power was compared to BMI and waist circumference using *z*-scores.

**Results:**

Of the study population, 5,605 participants had asthma (13.99% prevalence). After adjusting for possible confounders, the results showed that higher BRI was linked to greater asthma prevalence (OR = 1.41, 95% CI:1.27, 1.56, *p* < 0.0001). A J-shaped relationship between BRI and asthma prevalence (*p*-nonlinearity = 0) was found, with asthma prevalence rising significantly when BRI surpassed 4.34. BRI outperformed BMI and waist circumference in predicting asthma (BRI: OR = 1.180; BMI: OR = 1.169; W.C.: OR = 1.166). Subgroup and sensitivity analyses confirmed our results’ robustness.

**Conclusion:**

Adult asthma prevalence increases with increasing BRI levels, showing a J-shaped relationship. Keeping BRI under 4.34 is vital for lowering asthma prevalence, especially for overweight or obese individuals. In addition, BRI outperformed BMI and waist circumference in predicting asthma occurrence.

## Introduction

Asthma is a complex chronic respiratory disease whose primary symptoms include wheezing, coughing, and breathlessness. Nevertheless, the precise pathogenesis of asthma remains unclear (1, 2). According to the Global Initiative for Asthma (GINA) 2024 guidelines (3), approximately 300 million people worldwide are suffering from asthma, and it kills about 1,000 people every day. The World Health Organization (WHO) also predicts that the number of people with asthma could increase by 100 million by 2025 (4). Asthma not only adds to the global disease burden but also contributes to rising healthcare costs, loss of labor, and reduced ability to perform daily activities. Asthma affects adults in particular, potentially making chronic diseases like cardiovascular disease and diabetes more difficult to manage, and is especially common in older populations (5). While childhood asthma is usually associated with allergies (6), adult asthma can be caused by a variety of factors and often responds poorly to standard treatment regimens (7). Therefore, identifying the risk factors for adult asthma and taking preventive measures are crucial to improving patients’ quality of life and reducing healthcare expenditure.

The prevalence of obesity has increased rapidly in recent years. It is estimated that over one billion people around the world are affected by obesity (8). Body Mass Index (BMI) is commonly used to assess the degree of obesity in adults, with a BMI of 30 kg/m^2^ or more defined as obese. Numerous studies have confirmed that the risk of developing asthma increases with increasing obesity (9–12). BMI is a commonly used measure of obesity in studies exploring the relationship between obesity and asthma (13, 14). However, BMI does not reflect body fat distribution (15, 16). Therefore, more precise methods are needed to explore the relationship between obesity and asthma risk. Measures of body fat distribution have shown better predictive value than BMI in predicting diabetes and cardiovascular disease (17, 18). In order to reflect fat distribution more accurately, Thomas et al. (19) proposed a new anthropometric index, the Body Roundness Index (BRI). This index calculates body roundness based on an elliptical model of body shape. It uses eccentricity to estimate the percentage of visceral and total body fat. Unlike BMI, BRI considers waist circumference and provides a more complete picture of visceral fat distribution. Studies have shown that visceral fat has a greater effect on asthma development (20), so we hypothesized that BRI may be a better predictor of asthma incidence than BMI. BRI has a wide range of clinical applications. Previous studies have shown that the BRI has demonstrated superior predictive ability to traditional anthropometric methods in assessing the risk of diseases such as cardiometabolic diseases (21–23), kidney disease (24), and cancer (25), providing a more accurate tool for predicting and preventing obesity-related health problems in clinical practice. Until now, no studies have explicitly examined the association between the Body Roundness Index (BRI) and asthma.

To fill a knowledge gap about whether BRI is associated with prevalence in asthmatics, this study aims to analyze the data from the National Health and Nutrition Examination Survey (NHANES) in the United States from 2001 to 2018 and explore the relationship between BRI and asthma in U.S. adults, with the expectation of providing a new approach to the prevention and treatment of asthma.

## Materials and methods

### Data source and study population

The data used in this study originated from the 2001–2018 National Health and Nutrition Examination Survey (NHANES). This national survey assesses the nutritional and health status of adults and children in the U.S. The survey is unique in that it combines interviews and physical examinations and is published by the National Center for Health Statistics (NCHS). It uses a stratified multistage sampling design to produce a nationally representative sample of U.S. residents. The study was approved by the Research Ethics Review Board of the National Center for Health Statistics, and informed consent was obtained from all participants (26). For more information about the National Center for Health Statistics Institutional Review Board, please visit the website.^1^ Therefore, no other ethics committee approval was required for this study. The data from NHANES are publicly accessible.

**Figure 1:**
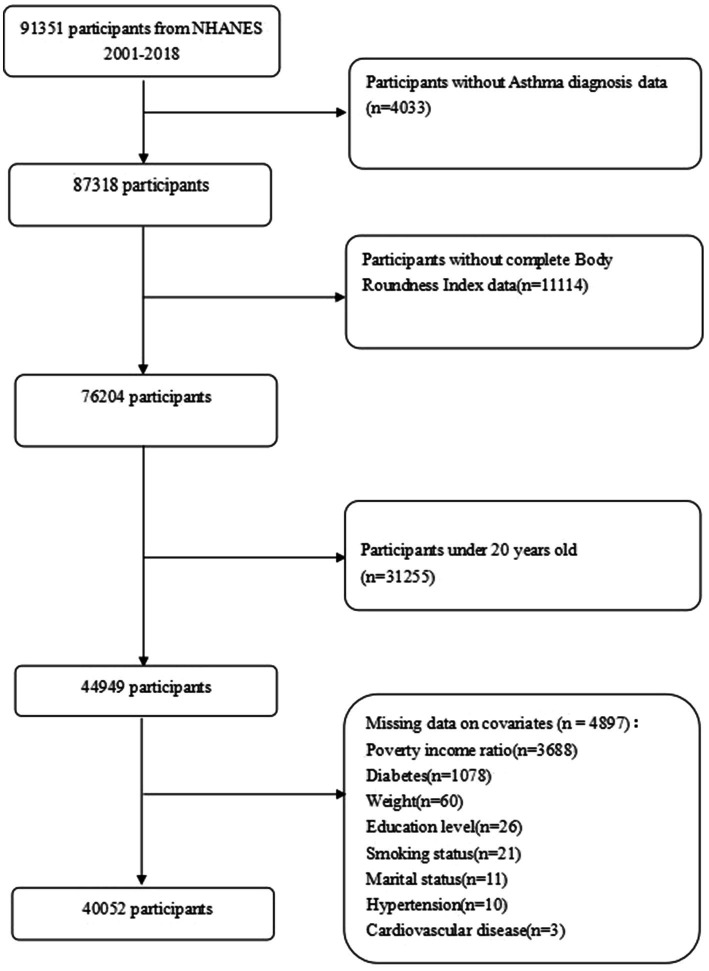
Cohort flow diagram in NHANES 2001–2018. NHANES, National Health and Nutrition Examination Survey.

Our analyses included participants with comprehensive information on asthma and the Body Roundness Index. Initially, a total of 91,351 participants were recruited. After excluding participants with no asthma diagnostic data (*n* = 4,033), lacking BRI data (*n* = 11,114), under 20 years of age (*n* = 31,255), and missing covariates (*n* = 4,897), our final analysis included 40,052 eligible participants, as shown in Figure 1.

### Definition of BRI

The Body Roundness Index (BRI) is calculated through the following formula: BRI = 364.2–365.5 × √ [1 – (W.C./(2π))2/ (0.5 × height)2] (19), where W.C. stands for the waist circumference. Both the waist circumference and the height are measured in meters. To guarantee data consistency and analysis accuracy, the height and waist circumference of the participants were measured at the mobile examination center using standardized procedures. Then, the BRI of each participant was calculated using these data. Based on the BRI values, the participants were divided into four quartile groups: Quartile 1 (1.05, 3.80); Quartile 2 (3.80, 5.06); Quartile 3 (5.06, 6.61); and Quartile 4 (6.61, 23.48). This classification method facilitates an in-depth study of the correlation between BRI levels and adult asthma prevalence in different populations.

### Assessment of the diagnosis of asthma

This study relied on patient self-reported diagnostic information from the NHANES database. The diagnostic criteria for asthma are based on data from questionnaires in the NHANES data about two questions: (1) “Has a doctor or other health professional told you that you have asthma?”; (2) “Do you still have asthma?” Only respondents who answered “yes” to both questions were diagnosed with asthma. In contrast, respondents who answered “no” to either question were not included as asthmatics (27–29). According to several peer-reviewed studies (30–32), self-reporting to diagnose asthma is highly accurate.

### Covariates

To reduce the potential bias, we have referred to previous studies (29, 33–37) and incorporated covariates such as age (20–39; 40–59; 60–79; 80+), gender (Male; Female), race (White; Black; Mexican American; Others), marital status (Married/Living with partner; Widowed/divorced/separated; Never married) and education level (Less than high school; High school; More than high school). The poverty income ratio (PIR) was divided into Low (PIR <1.3), Middle (PIR 1.3–3.5), and High (PIR >3.5). The number of cigarettes smoked is used as a measure of an individual’s smoking status. Smoking status is classified into three categories: (1) never smokers, defined as individuals who have not smoked more than 100 cigarettes; (2) former smokers, comprising those who have smoked more than 100 cigarettes but have ceased smoking; and (3) current smokers, including those who have smoked up to 100 cigarettes and continue to smoke. The diagnosis of diabetes is based on the following criteria: whether the subject is using insulin, has doctor-diagnosed diabetes, or is taking medication for blood sugar control. A subject is recognized as having diabetes if they test positive for any of the above. The diagnosis of cardiovascular disease requires a positive diagnosis by a physician based on a history of myocardial infarction, angina pectoris, coronary heart disease, or stroke. Criteria for determining hypertension included that the patient had a documented diagnosis of hypertension at each of their multiple visits to the doctor, that the doctor had recommended that they take antihypertensive medication, or that the average of three consecutive blood pressure measurements was not less than 140 mm Hg for systolic blood pressure and 90 mm Hg for diastolic blood pressure.

### Statistical analysis

To correct possible bias caused by oversampling, this study followed the NHANES guidelines and used a sample weighting method. Based on the NHANES guidelines for analysis, we divided the weights for each 2-year cycle by 9 to generate new sample weights for the combined survey cycle. In descriptive analyses, we reported means and standard deviations (S.D.) for baseline characteristics of continuous variables. In contrast, categorical variables were presented as proportions. Student’s *t*-test assessed differences in means of continuous variables. Differences in the proportions of categorical variables were rated using Pearson’s chi-square test. We used multivariate logistic regression models to analyze the relationship between different Body Roundness Index (BRI) levels and adult asthma prevalence. Model 1 was not adjusted for covariates. In Model 2, we adjusted for age, gender, race, marital status, education level, and PIR. In Model 3, we additionally adjusted for smoking status, diabetes, cardiovascular disease, and hypertension based on Model 2. To verify the stability of the findings, we classify the continuous variable BRI into categorical variables (quartiles). To deal with the possible nonlinear relationship between BRI and asthma risk, we used generalized additive modeling (GAM) and smoothed curve fitting techniques. In addition, we used segmented linear regression models, also known as two-stage linear regression models, to fit stages in the data and identify threshold effects in nonlinear relationships. When nonlinear associations were found, we identified threshold effects in each stage through the segmented linear regression model. We applied a two-step recursive approach to finding the optimal breakpoint *K* value, which was determined based on the model that best produced maximum likelihood. To facilitate the comparison of BRI with other obesity indicators in terms of the odds ratio, we standardized these indicators, calculated their *z*-scores, and included them in the linear model analysis. Finally, we conducted subgroup and sensitivity analyses to ensure the results’ reliability. All statistical analyses used Empower software^2^ (X&Y Solutions, Inc., Boston, MA) and R version 4.2.3^3^ (R Foundation). In assessing the statistical significance of the results, we used the criterion of a *p*-value of less than 0.05.

## Results

### Baseline of participants

Table 1 demonstrates the demographics and characteristics of the study participants. A total of 40,052 participants were included in this study, of which 5,605 (13.99%) had asthma. Participants ranged from 20 to 59 (78.43%) and women (58.55%). Of all participants, 71.26% were Non-Hispanic White. The asthma population tended to be younger, female, Non-Hispanic White, married or living with a partner, with a higher education level and higher income, and former or never smokers. In addition, asthmatics had relatively lower rates of diabetes, cardiovascular disease, and hypertension. Notably, the asthma group had significantly higher Body Roundness Index, Body Mass Index, waist circumference, and weight than the non-asthma group (*p* < 0.0001).

**Table 1:** Baseline characteristics of the study population.

Characteristics	Total (*n* = 40,052)	Non-Asthma (*n* = 34,447)	Asthma (*n* = 5,605)	*p*-value
Body Roundness Index	5.27 (0.03)	5.20 (0.02)	5.64 (0.06)	< 0.0001
Body Mass Index (Kg/m2)	28.77 (0.07)	28.58 (0.07)	29.88 (0.16)	< 0.0001
Waist circumference (cm)	98.57 (0.18)	98.23 (0.17)	100.52 (0.39)	< 0.0001
Weight (Kg)	82.29 (0.20)	81.89 (0.19)	84.68 (0.47)	< 0.0001
Age, years				< 0.0001
20–39	13,445 (37.19)	11,304 (36.34)	2,141 (42.12)	
40–59	13,395 (38.71)	11,573 (39.12)	1822 (36.31)	
60–79	10,796 (20.48)	9,373 (20.68)	1,423 (19.32)	
80+	2,416 (3.62)	2,197 (3.86)	219 (2.25)	
Gender, %				< 0.0001
Male	19,948 (49.01)	17,554 (50.30)	2,394 (41.45)	
Female	20,104 (50.99)	16,893 (49.70)	3,211 (58.55)	
Race, %				< 0.0001
Others	7,084 (12.05)	6,131 (12.09)	953 (11.84)	
Mexican American	6,457 (7.91)	5,924 (8.47)	533 (4.67)	
Black	8,397 (10.85)	7,044 (10.61)	1,353 (12.22)	
White	18,114 (69.18)	15,348 (68.83)	2,766 (71.26)	
Marital status, %				< 0.0001
Never married	7,031 (17.62)	5,845 (17.07)	1,186 (20.84)	
Widowed/divorced/separated	8,833 (18.22)	7,450 (17.87)	1,383 (20.27)	
Married/Living with a partner	24,188 (64.16)	21,152 (65.06)	3,036 (58.88)	
Education level, %				0.001
< High school	9,969 (15.89)	8,740 (16.12)	1,229 (14.54)	
High school	9,329 (23.83)	8,084 (24.12)	1,245 (22.12)	
>High school	20,754 (60.29)	17,623 (59.77)	3,131 (63.34)	
Poverty income ratio, %				< 0.0001
Low (≤1.3)	12,276 (21.01)	10,276 (20.21)	2000 (25.69)	
Middle (>1.3and ≤ 3.5)	15,262 (35.90)	13,307 (36.22)	1955 (34.04)	
High (>3.5)	12,514 (43.09)	10,864 (43.57)	1,650 (40.27)	
Smoking status, %				< 0.0001
Never	21,557 (53.77)	18,819 (54.45)	2,738 (49.82)	
Former	9,900 (24.72)	8,436 (24.52)	1,464 (25.91)	
Now	8,595 (21.50)	7,192 (21.03)	1,403 (24.28)	
Diabetes, %				< 0.001
No	34,869 (90.57)	30,153 (90.87)	4,716 (88.87)	
Yes	5,183 (9.43)	4,294 (9.13)	889 (11.13)	
Cardiovascular disease, %				< 0.0001
No	35,699 (91.68)	30,959 (92.30)	4,740 (88.09)	
Yes	4,353 (8.32)	3,488 (7.70)	865 (11.91)	
Hypertension, %				< 0.0001
No	23,196 (63.23)	20,174 (63.78)	3,022 (59.97)	
Yes	16,856 (36.77)	14,273 (36.22)	2,583 (40.03)	

### A higher BRI index is associated with higher asthma prevalence

Table 2 demonstrates the correlation between BRI and asthma. Our data analysis revealed that an increase in BRI was associated with an increase in asthma risk. Models 1, 2, and 3 showed a positive association between BRI and asthma risk. After fully adjusting for covariates, we found that for every one-unit increase in BRI, participants were 7% more likely to develop asthma (Model 3: OR = 1.07, 95% CI: 1.06–1.09). This positive correlation remained statistically significant when the BRI was further analyzed by dividing it into quartiles. Participants with a BRI in the highest quartile had a 41% higher risk of developing asthma than those with a BRI in the lowest quartile (OR = 1.41, 95% CI: 1.27–1.56; *p* for trend <0.0001).

**Table 2:** The relationship between Body Roundness Index and Asthma prevalence among participants from the NHANES 2001–2018.

Characteristic	Model 1 OR (95% CI) *p*-value	Model 2 OR (95% CI) *p*-value	Model 3 OR (95% CI) *p*-value
Continuous	1.08 (1.06, 1.10) <0.0001	1.09 (1.07, 1.11) <0.0001	1.07 (1.06, 1.09) <0.0001
Categories			
Quartile 1 (1.05, 3.80)	Reference	Reference	Reference
Quartile 2 (3.80, 5.06)	0.81 (0.73, 0.91) <0.001	0.93 (0.83, 1.04) 0.20	0.91 (0.82, 1.02) 0.12
Quartile 3 (5.06, 6.61)	0.88 (0.79, 0.99) 0.03	1.05 (0.93, 1.18) 0.41	1.00 (0.89, 1.12) 0.98
Quartile 4 (6.61, 23.48)	1.41 (1.27, 1.55) <0.0001	1.56 (1.41, 1.73) <0.0001	1.41 (1.27, 1.56) <0.0001
*p* for trend	<0.0001	<0.0001	<0.0001

Model 1 was adjusted for no covariates.

Model 2 was adjusted for age, gender, race, marital status, education level, and poverty income ratio.

Model 3 was adjusted for age, gender, race, marital status, education level, poverty income ratio, smoking status, diabetes, cardiovascular disease, and hypertension.

OR, Odds Ratio; CI, Confidence Interval.

**Figure 2:**
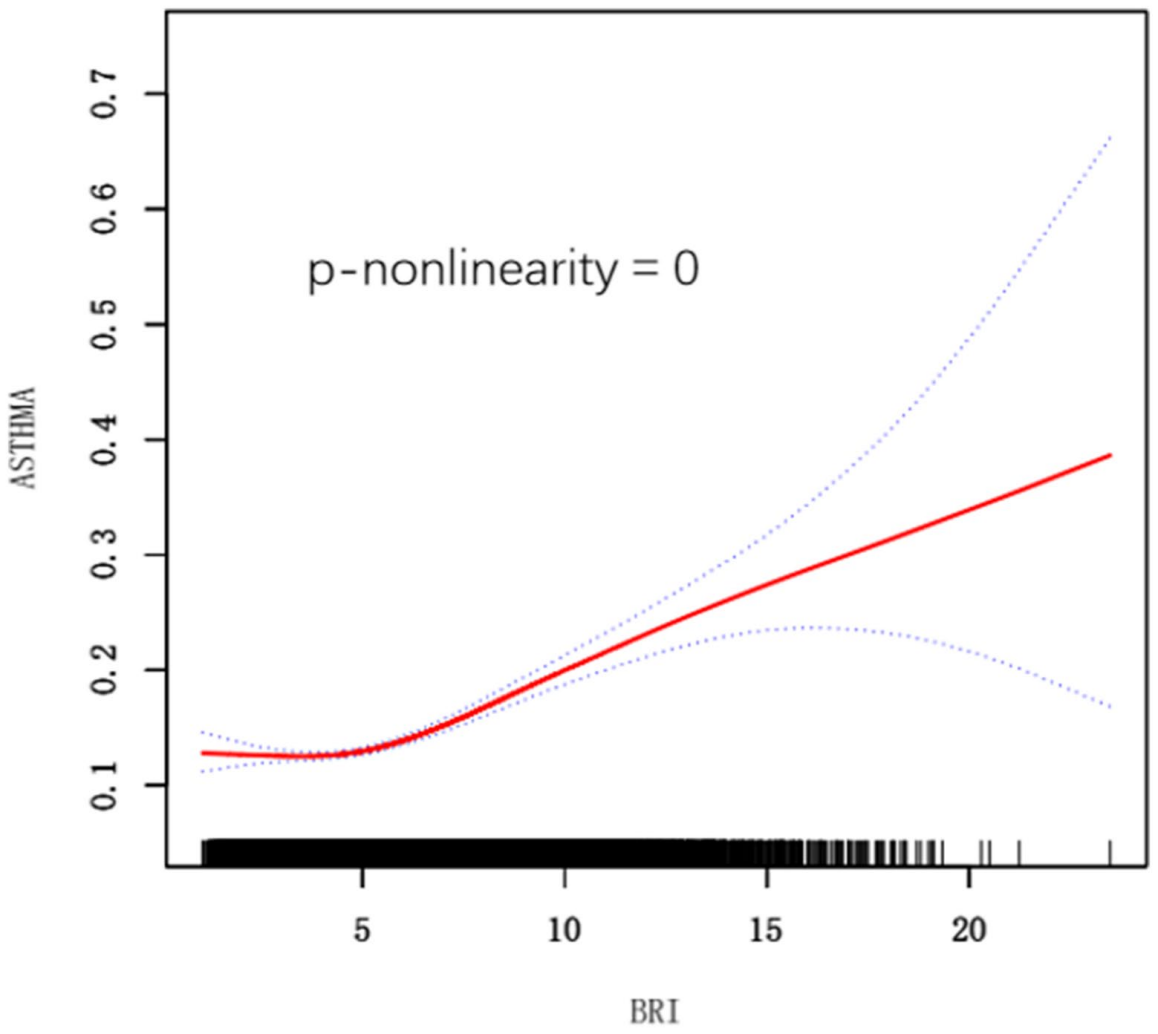
Smooth curve fitting for BRI and Asthma. BRI, Body Roundness Index. Adjusted for age, gender, race, marital status, education level, poverty income ratio, smoking status, diabetes, cardiovascular disease, and hypertension.

In addition, we used a smoothed curve-fitting technique to delve deeper into the association between BRI and asthma risk, and our analysis revealed a positive nonlinear association (Figure 2).

### Body roundness index showed a stronger correlation than other markers of obesity, including W.C. and body mass index for asthma

**Figure 3:**
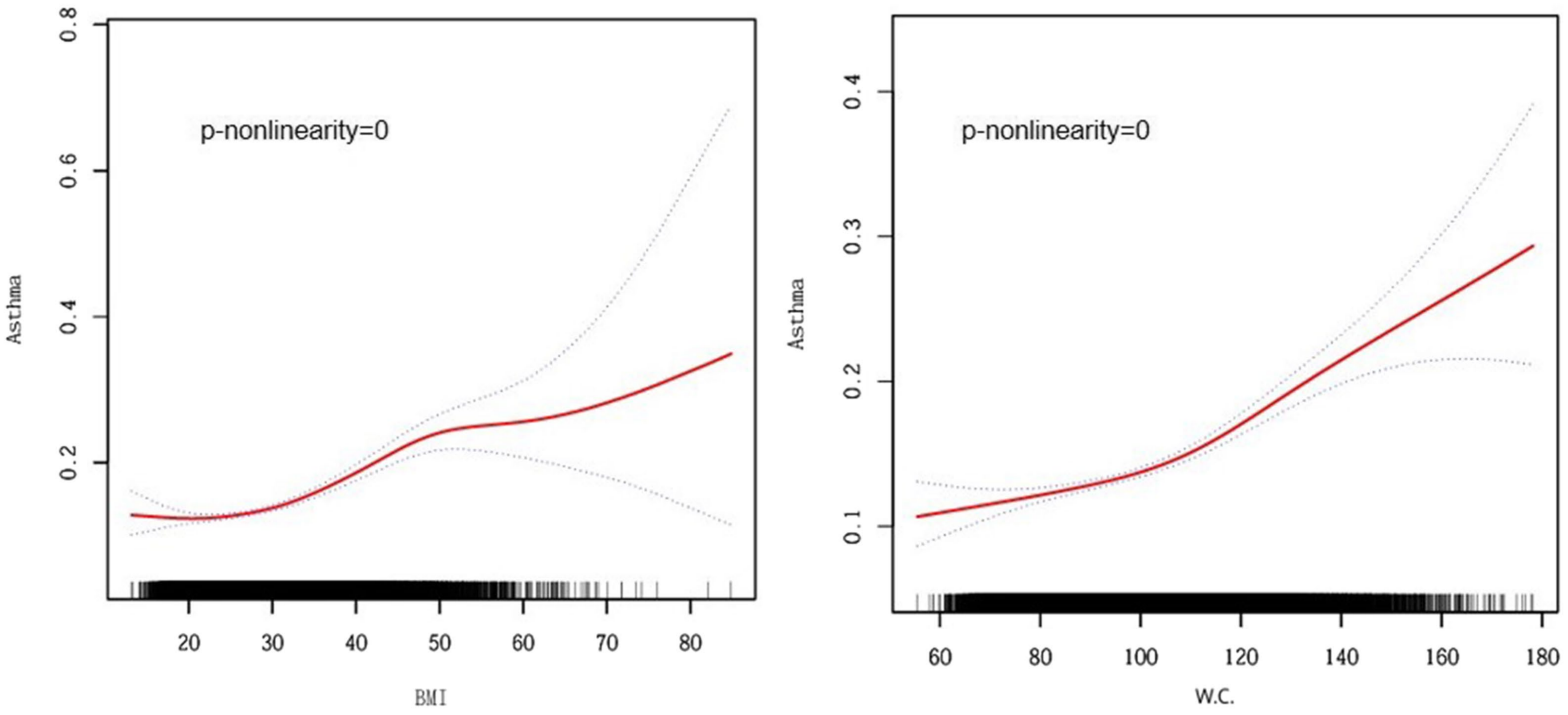
Smooth curve fitting for BMI, W.C., and Asthma. BMI, Body Mass Index, W.C., Waist Circumference. Adjusted for age, gender, race, marital status, education level, poverty income ratio, smoking status, diabetes, cardiovascular disease, and hypertension.

Smoothed curve analyses revealed nonlinear associations between obesity indicators other than BRI (e.g., BMI and waist circumference) and asthma risk (Figure 3). Following this, we applied a segmented regression model to fit the individual data intervals and assessed the threshold effect accordingly (Table 3).

**Table 3:** Threshold effect analysis of BRI, BMI, and W.C. on Asthma.

	BRI	BMI	W.C.
Fitting by the standard linear model			
OR (95% CI), *p*-value	1.083 (1.069, 1.096) <0.0001	1.027 (1.023, 1.031) <0.0001	1.011 (1.009, 1.013) <0.0001
Fitting by two-piecewise linear model			
Breakpoint (K)	4.34	21.02	106.90
OR1 (<K)	0.957 (0.910, 1.006) 0.0851	0.933 (0.885, 0.982) 0.0086	1.007 (1.004, 1.010) <0.0001
OR2 (>K)	1.107 (1.091, 1.124) <0.0001	1.029 (1.025, 1.034) <0.0001	1.016 (1.012, 1.019) <0.0001
OR2/OR1	1.157	1.103	1.009
Logarithmic likelihood ratio test *p*-value	<0.001	<0.001	0.002

Adjusted for age, gender, race, marital status, education level, poverty income ratio, smoking status, diabetes, cardiovascular disease, and hypertension.

OR, Odds Ratio; CI, Confidence Interval; BRI, Body Roundness Index; BMI, Body Mass Index; W.C., Waist Circumference.

To compare the OR of BRI with other obesity indicators, we standardized these indicators, calculated their z-scores, and included them in a linear model analysis (Table 4). The results showed that BRI was more significantly associated with asthma risk than other obesity indicators such as Body Mass Index (BMI) and Waist Circumference (W.C.) (BRI: OR = 1.180; BMI: OR = 1.169; W.C.: OR = 1.166), which suggests that BRI may be a superior predictor of asthma risk.

**Table 4:** Associations between the *z*-scores of markers of obesity and Asthma prevalence.

OR (95% CI), *p*-value			
*Z*-scores	BRI	BMI	W.C.
Continuous	1.180 (1.136, 1.226) <0.000001	1.169 (1.128, 1.212) <0.000001	1.166 (1.123, 1.211) <0.000001

Adjusted for age, gender, race, marital status, education level, poverty income ratio, smoking status, diabetes, cardiovascular disease, and hypertension.

OR, Odds Ratio; CI, Confidence Interval; BRI, Body Roundness Index; BMI, Body Mass Index; W.C., Waist Circumference.

### Subgroup and sensitivity analysis

**Figure 4:**
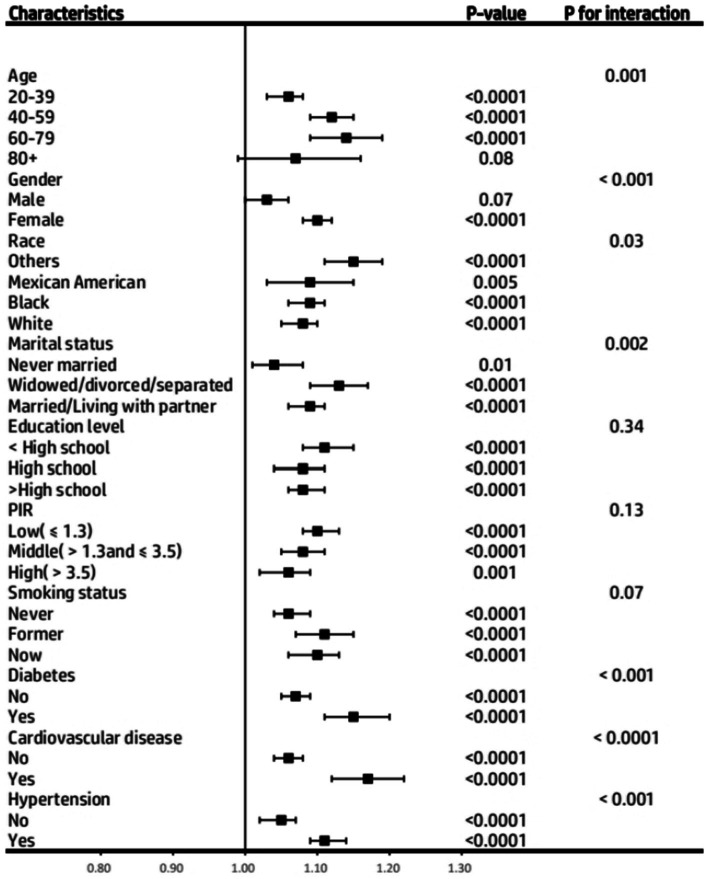
Subgroup analysis of the association between Body Roundness Index and Asthma prevalence among participants from the NHANES 2001–2018. PIR, Poverty Income Ratio.

Data from subgroup analyses revealed a consistent trend: the positive correlation between BRI and asthma risk remained stable in subjects with various characteristics. However, the more significant population characteristics were 60–79 years old, women, other races, widowed/divorced/separated, diabetes, cardiovascular disease, and hypertension (Figure 4). To avoid potential selection bias caused by the large number of missing values in the covariates of diabetes and the poverty income ratio, in the sensitivity analysis, we excluded these two variables from the analysis and re-included the study population. Subsequently, we again used the weighted multivariate logistic regression model to assess the association between BRI levels and the onset of asthma. The analysis results were consistent with the results shown in Table 5, verifying the stability of our research results.

**Table 5:** The relationship between the Body Roundness Index and Asthma prevalence among participants from the NHANES 2001–2018 (Covariates did not include poverty income ratio and diabetes).

Characteristic	Model 1 OR (95% CI) *p*-value	Model 2 OR (95% CI) *p*-value	Model 3 OR (95% CI) *p*-value
Continuous	1.08 (1.06, 1.10) <0.0001	1.09 (1.08, 1.11) <0.0001	1.08 (1.06, 1.10) <0.0001
Categories			
Quartile 1 (0.94, 3.82)	Reference	Reference	Reference
Quartile 2 (3.82, 5.09)	0.81 (0.73, 0.90) <0.0001	0.93 (0.84, 1.03) 0.17	0.91 (0.82, 1.02) 0.09
Quartile 3 (5.09, 6.63)	0.90 (0.81, 1.01) 0.07	1.07 (0.96, 1.20) 0.23	1.02 (0.91, 1.15) 0.72
Quartile 4 (6.63, 23.48)	1.40 (1.28, 1.53) <0.0001	1.58 (1.44, 1.74) <0.0001	1.44 (1.30, 1.58) <0.0001
*p* for trend	<0.0001	<0.0001	<0.0001

Model 1 was adjusted for no covariates.

Model 2 was adjusted for age, gender, race, marital status, and education level.

Model 3 was adjusted for age, gender, race, marital status, education level, smoking status, cardiovascular disease, and hypertension.

OR: Odds Ratio, CI: Confidence Interval.

## Discussion

This study is the first to quantitatively analyze the relationship between Body Roundness Index (BRI) and asthma in U.S. adults. After adjusting for relevant covariates and based on cross-sectional data from the NHANES from 2001 to 2018, we used weighted logistic regression analysis to find that higher BRI was associated with an increased risk of asthma in adults. The findings suggest a J-shaped association between BRI and asthma risk, where changes in BRI have little effect on asthma risk below a certain threshold. However, once that threshold is exceeded, asthma risk rises as BRI increases. We found that the critical tipping point was 4.34. In addition, we found that BRI was more effective in predicting asthma risk than traditional obesity indicators such as BMI and waist circumference. Subgroup analyses suggest that management strategies for obese populations identified based on BRI should focus specifically on patients 60–79 years of age, women, nonwhite race, divorced/widowed/separated, diabetes, cardiovascular disease, and hypertension. These findings may lead to the development of new clinical guidelines.

Primary asthma prevention has been a challenge in research and clinical practice compared to timely and effective treatment options (38). The link between asthma and obesity is particularly complex, with obese asthmatics more likely to have persistent symptoms, increased absenteeism, increased medication use, and other related problems. Moreover, obese asthmatics are less likely to experience symptom relief and are more likely to develop severe persistent asthma (39). However, accurately assessing the actual extent to which obesity affects asthma is also a challenge. Although Body Mass Index (BMI) is a commonly used measure of obesity and is easy to measure, it has limitations. For example, a study of more than 40,000 U.S. adults by Tomiyama et al. found that half of the overweight population and nearly one-third of the obese population were metabolically healthy. In contrast, over 30% of the normal-weight population had metabolic abnormalities (40). The prevailing view is that the distribution of body fat and body composition cannot be described by BMI alone and that significant differences exist even among individuals with the same BMI (41). Prillaman suggests that the definition of obesity needs to be updated (42). He argues that BMI ignores essential factors such as visceral fat, which may significantly impact health. Therefore, in this study, we used the Body Roundness Index (BRI) to assess the association between obesity and asthma in adults more accurately.

Obesity can be categorized as visceral obesity, subcutaneous fat accumulation in the trunk and extremities, and fat ectopic in skeletal muscle (43). Visceral adipose tissue, in particular, being metabolically active, produces a variety of cytokines that are negatively correlated with one-second forceful expiratory volume (FEV1), FEV1% prediction, and forced lung capacity (FVC) (43, 44). Studies have noted an association between visceral adipose tissue (VAT) and the development of asthma and decreased lung function in adolescents and adults (20), whereas peripheral obesity has not been significantly associated with these health problems. A large-scale study conducted in Vienna found that a significantly higher proportion of asthmatics were obese compared to controls. There was a significant increase in visceral adipose tissue (VAT), especially in adult and elderly asthmatics (45). Indicators of abdominal obesity, such as waist circumference and waist-to-height ratio, are positively correlated with the prevalence of asthma. However, these indicators do not accurately reflect visceral adiposity (9, 46–49). Although magnetic resonance imaging (MRI) and abdominal computed tomography (C.T.) can assess visceral fat, both methods are costly and not easily accessible (44, 50). In contrast, the Body Roundness Index (BRI) is less costly and more accessible to patients.

The mechanisms linking asthma and visceral adipose tissue are not fully understood. Possible pathways of association include: (1) Adipose tissue may secrete adipokines such as leptin and adiponectin, as well as cytokines such as IL-6, IL-8, and TNFα, which triggers an inflammatory response, which in turn may lead to airway inflammation and asthma (51). (2) Airway hyperreactivity may be induced by the release of adipokines into serum during adipose tissue inflammation (52). (3) The link between obesity and asthma may also include chronic low-grade systemic inflammation, insulin resistance, and dyslipidemia (51). Studies have shown that visceral adipose tissue is more likely to be associated with adverse inflammation, blood glucose, and lipid levels than subcutaneous adipose tissue (53). Visceral fat, not subcutaneous fat, is associated with asthma (54). Further studies have also confirmed that abnormalities in visceral fat are associated with decreased lung function and increased inflammation in patients with obesity-related asthma (55). Increased visceral fat is associated with elevated levels of IL-6 (56, 57), which can disrupt the balance of fatty acid metabolism and trigger an active inflammatory state in the body (58, 59). Researchers have realized the link between IL-6 and the development of asthma (60). These findings support the general view that visceral fat is associated with a more active inflammatory state in the body. This view provides a plausible explanation for our findings: adults with a higher Body Roundness Index (BRI) are more likely to have more visceral fat, which triggers a robust inflammatory response that increases their risk of developing asthma.

This study has several strengths. First, the NHANES survey conducted between 2001 and 2018 used scientific sampling methods based on a representative sample of the U.S. general population and followed a rigorous study design with comprehensive quality control measures to ensure the reliability and scientific validity of the data. Second, the study meticulously adjusted for socioeconomic status, co-morbidities, and other possible confounders based on clinical knowledge and historical research, further ensuring the robustness of the findings. Finally, to compare the correlation between BRI and other obesity indicators, the corresponding Z-scores were calculated and included in the linear model analysis, thus ensuring the accuracy of the results. The study results showed that the correlation between BRI and asthma exceeded that of traditional obesity indicators such as waist circumference and BMI. Therefore, from a public health perspective, BRI, as a non-invasive and easy-to-administer screening tool, effectively assesses asthma risk in adults.

However, this study has its limitations. Firstly, given the study’s cross-sectional nature, we were unable to establish a causal association between BRI and asthma in adults, and future studies will need to validate this relationship with the help of cohort studies. Second, the study’s results may apply to specific countries and ethnic groups, and their generalizability has yet to be verified. Differences in fat distribution in different populations due to genetic and environmental factors may lead to differences in fat distribution, which in turn may affect the accuracy of BRI in predicting asthma prevalence. Therefore, future studies should include multinational and multiethnic samples to validate our findings further. Finally, the lack of detailed clinical variables in the database, such as medication history or specific type of asthma, is missing information that limits the depth of the study and needs to be supplemented by further research. Despite its limitations, this study demonstrated its value by revealing an association between BRI and asthma in adults. It was particularly innovative in exploring this relationship using National Health and Nutrition Examination Survey (NHANES) data.

In sum, our findings support an association between Body Roundness Index (BRI) and adult asthma risk, where higher BRI values may predict an increased risk of asthma in adults. This finding is essential for deepening our understanding of the relationship between obesity and asthma. It suggests that BRI has the potential to serve as an indicator of asthma prevention. Future studies need to further validate the efficacy of the BRI as a predictive tool for assessing adult asthma risk in different countries and ethnicities to expand its applicability to the population.

## Data Availability

Publicly available datasets were analyzed in this study. This data can be found here: all data are freely available in NHANES. This data can be found at: https://www.cdc.gov/nchs/nhanes/index.htm.

## Footnotes

^1^ http://www.cdc.gov/nchs/nhanes/irba98.htm

^2^ www.empowerstats.com

^3^ http://www.R-project.org
